# Characterization in Effective Stimulation on the Magnitude, Gating, Frequency Dependence, and Hysteresis of *I*_Na_ Exerted by Picaridin (or Icaridin), a Known Insect Repellent

**DOI:** 10.3390/ijms23179696

**Published:** 2022-08-26

**Authors:** Ai-Li Shiau, Chih-Szu Liao, Chi-Wen Tu, Sheng-Nan Wu, Hsin-Yen Cho, Meng-Cheng Yu

**Affiliations:** 1 Ditmanson Medical Foundation Chia-Yi Christian Hospital, Chiayi City 60002, Taiwan; 2 Department of Physiology, National Cheng Kung University Medical College, Tainan 70101, Taiwan; 3 Institute of Basic Medical Sciences, National Cheng Kung University Medical College, Tainan 70101, Taiwan

**Keywords:** picaridin (icaridin), voltage-gated Na^+^ current, late Na^+^ current, transient Na^+^ current, persistent Na^+^ current, resurgent Na^+^ current, current kinetics, voltage-dependent hysteresis

## Abstract

Picaridin (icaridin), a member of the piperidine chemical family, is a broad-spectrum arthropod repellent. Its actions have been largely thought to be due to its interaction with odorant receptor proteins. However, to our knowledge, to what extent the presence of picaridin can modify the magnitude, gating, and/or the strength of voltage-dependent hysteresis (Hys_(V)_) of plasmalemmal ionic currents, such as, voltage-gated Na^+^ current [*I*_Na_], has not been entirely explored. In GH_3_ pituitary tumor cells, we demonstrated that with exposure to picaridin the transient (*I*_Na(T)_) and late (*I*_Na(L)_) components of voltage-gated Na^+^ current (*I*_Na_) were differentially stimulated with effective EC_50_’s of 32.7 and 2.8 μM, respectively. Upon cell exposure to it, the steady-state current versus voltage relationship *I*_Na(T)_ was shifted to more hyperpolarized potentials. Moreover, its presence caused a rightward shift in the midpoint for the steady-state inactivate curve of the current. The cumulative inhibition of *I*_Na(T)_ induced during repetitive stimuli became retarded during its exposure. The recovery time course from the *I*_Na_ block elicited, following the conditioning pulse stimulation, was satisfactorily fitted by two exponential processes. Moreover, the fast and slow time constants of recovery from the *I*_Na_ block by the same conditioning protocol were noticeably increased in the presence of picaridin. However, the fraction in fast or slow component of recovery time course was, respectively, increased or decreased with an increase in picaridin concentrations. The Hys_(V)_’s strength of persistent *I*_Na_ (*I*_Na(P)_), responding to triangular ramp voltage, was also enhanced during cell exposure to picaridin. The magnitude of resurgent *I*_Na_ (*I*_Na(R)_) was raised in its presence. Picaritin-induced increases of *I*_Na(P)_ or *I*_Na(R)_ intrinsically in GH_3_ cells could be attenuated by further addition of ranolazine. The predictions of molecular docking also disclosed that there are possible interactions of the picaridin molecule with the hNa_V_1.7 channel. Taken literally, the stimulation of *I*_Na_ exerted by the exposure to picaridin is expected to exert impacts on the functional activities residing in electrically excitable cells.

## 1. Introduction

Picaridin (icaridine, 1-piperidinecarboxylic acid 2-(2-hydroxyethyl)-1-methylpropylester), a cyclic amine and a member of the piperidine chemical family, is viewed as a synthetic, broad-spectrum arthropod repellent [1,2,3,4,5,6,7,8,9,10,11,12,13,14,15,16]. The repellent and deterrent activities of picaridin have been previously demonstrated to involve olfactory sensing in mosquitoes and ticks, via their interactions with odorant receptor proteins residing in neurons [17,18,19,20,21,22]. However, to the best of our knowledge, the questions of whether and how picaridin, or other relevant compounds, act to cause any modifications on other types of transmembrane ionic currents have not yet been answered.

It has been established that nine isoforms (i.e., Na_V_1.1–1.9 [or SCN1A-SCN5A and SCN8A-SCN11A] of voltage-gated Na^+^ (Na_V_) channels are distributed in mammalian excitable tissues which are present in the central or peripheral nervous system, as well as in the neuroendocrine system [23,24,25]. Once activated, the increased activity of these channels can quickly depolarize the cell membrane and, in turn, elicit the upstroke of the action potential, thereby intrinsically governing the amplitude, frequency, and/or pattern of firing action potentials in an array of excitable cells [23,24,25,26,27]. Of additional note, some inhibitors of Na_V_ channels (e.g., KMUP-1 and ranolazine) were demonstrated to increase the inactivation rate of *I*_Na_ [28,29,30], while some activations of Na_V_ channels (e.g., tefluthrin) could preferentially retard the inactivation rate, as well as enhance the transient (*I*_Na(T)_) and late (*I*_Na(L)_) components of *I*_Na_ [31,32]. KMUP-1 was viewed as a xanthine and piperazine derivative [30], and ranolazine as a piperazine derivative, which is structurally similar to trimetazidine and known to be a blocker of *I*_Na(L)_ [28,29,33,34], while tefluthrin has been reported to be a synthetic type-I pyrethroid used to kill a wide range of insects [35,36]. However, whether and how picaridin can affect the magnitude, gating kinetics, and/or voltage-dependent hysteresis (Hys_(V)_) of *I*_Na_ remains mostly obscure, although it is an insect repellent which belongs to the piperidine chemical family.

Due to the reasons elaborated above, the overall objective of the current investigation was to explore if picaridin, and other relevant compounds, could exert any adjustments on the magnitude, gating, use dependence, and/or Hys_(V)_ behavior of plasmalemmal ionic currents, particularly voltage-gated Na^+^ current (*I*_Na_). The biophysical and pharmacological properties of *I*_Na_, including transient *I*_Na_ (*I*_Na(T)_), late *I*_Na_ (*I*_Na(L)_), persistent *I*_Na_ (*I*_Na(P)_) and resurgent *I*_Na_ (*I*_Na(R)_), were extensively studied in pituitary GH_3_ lactotrophs. Findings from the present studies provide the notion that picaridin is capable of perturbing the magnitude, gating, and/or Hys_(V)_ strength of *I*_Na(T)_ in concentration-, time-, frequency- and Hys_(V)_-dependent manners.

## 2. Results

### 2.1. Stimulatory Effect of Picaridin on Voltage-Gated Na^+^ Current (I_Na_) Experimentally Recorded from Pituitary GH_3_ Cells

**Figure 1:**
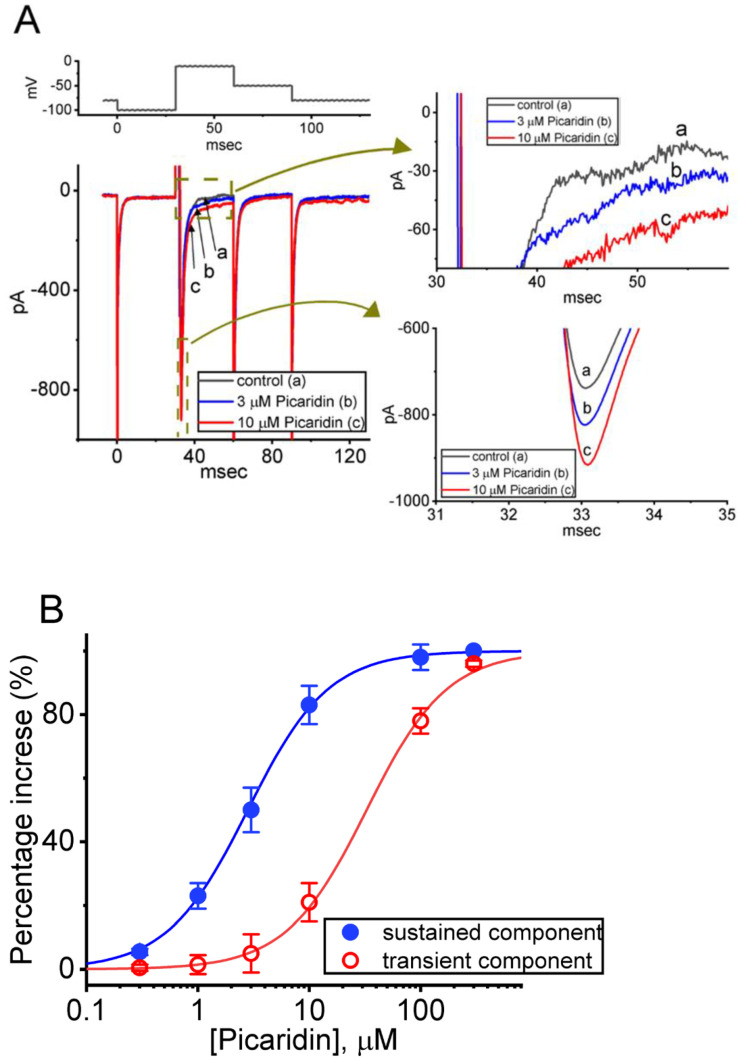
Stimulatory effect of picaridin on voltage-gated Na^+^ current (*I*_Na_) measured from pituitary GH_3_ cells. In this set of measurements, we placed cells in Ca^2+^-free Tyrode’s solution containing 0.5 mM CdCl_2_ and 10 mM tetraethylammonium chloride (TEA), and the recording pipette was filled up with a Cs^+^-enriched solution. (**A**) Exemplar current traces evoked by a 30-ms depolarizing pulse from −100 to −10 mV (indicated in the uppermost part (**A**)). a: control (i.e., picaridin was not present); b: 3 μM picaridin; and c: 10 μM picaridin. The right side shows the expanded records with curve arrows from the brown dashed boxes in the left side. (**B**) Concentration-dependent stimulation of transient (peak, *I*_Na(T)_, red open circles) or sustained (late, *I*_Na(L)_, blue filled circles) caused by picaridin. The amplitude of *I*_Na(T)_ or I_Na(L)_ was taken at the start- or end-pulse of the depolarizing command voltage from −100 to −10 mV for a duration of 30 ms. Each point represents the mean ± SEM (n = 8). The sigmoidal curves, on which the data points are overlaid, were reliably fitted to a modified Hill equation, as detailed under Materials and Methods.

In the start of experiments, we attempted to explore if cell exposure to picaridin produced any modifications on the magnitude of *I*_Na_ identified in these cells. To verify the appearance of *I*_Na_, we used Ca^2+^-free Tyrode’s solution as a bathing medium in which 10 mM tetraethylammonium chloride (TEA) and 0.5 mM CdCl_2_ were contained, and the recording electrode was then filled up with a Cs^+^-containing solution. As illustrated in Figure 1A, one minute after cells were exposed to picaridin (3 or 10 μM), the *I*_Na_ magnitude (with a rapidly activating and inactivating property) elicited by 30-ms depolarizing step from −100 to −10 mV became progressively increased. For example, one minute after cell exposure to 3 or 10 μM, the peak (or transient, *I*_Na(T)_) component of *I*_Na_ was respectively raised to 828 ± 44 pA (n = 8, *p* < 0.05) or 900 ± 50 pA (n = 8, *p* < 0.05) from a control value of 712 ± 39 pA (n = 8, *p* < 0.05). The observed picaridin-mediated increase of *I*_Na(T)_ was also concurrently accompanied by a striking retardation in the inactivation time course of the current, as evidenced by a striking increase in the value of the slow component of the inactivation time constant (τ_inact(S)_) from 12 ± 2 to 19 ± 4 ms (n = 8, *p* < 0.05). After washout of picaridin, current magnitude returned to 719 ± 41 pA (n = 8, *p* < 0.05). Moreover, picaridin-mediated stimulation of *I*_Na_ was also counteracted by further addition of tetrodotoxin (TTX, 1 μM) or ranolazine (Ran, 10 μM). TTX or Ran was reported to suppress *I*_Na_ in pituitary lactotrophs [26,28,37].

The relationship between the picaridin concentration and the percentage increase of *I*_Na(T)_ or *I*_Na(L)_ was further constructed (Figure 1B). Addition of picaridin was noticed to increase the *I*_Na(T)_ or *I*_Na(L)_ amplitude in a concentration-dependent fashion. When the experimental data became least-squares fitted to a Hill function, as detailed in Materials and Methods, the half-maximal concentration (i.e., EC_50_) required for the stimulatory effect of picaridin on *I*_Na(T)_ or *I*_Na(L)_ observed in these cells was yielded as 32.7 or 2.8 μM, respectively. Overall, the data from these experiments allowed us to ascertain that the presence of picaridin caused a stimulatory but differential effect on *I*_Na(T)_ and *I*_Na(L)_ in a concentration-dependent manner.

### 2.2. Effect of Picaridin on Mean Current versus Voltage (I–V) Relationship of I_Na(T)_

**Figure 2:**
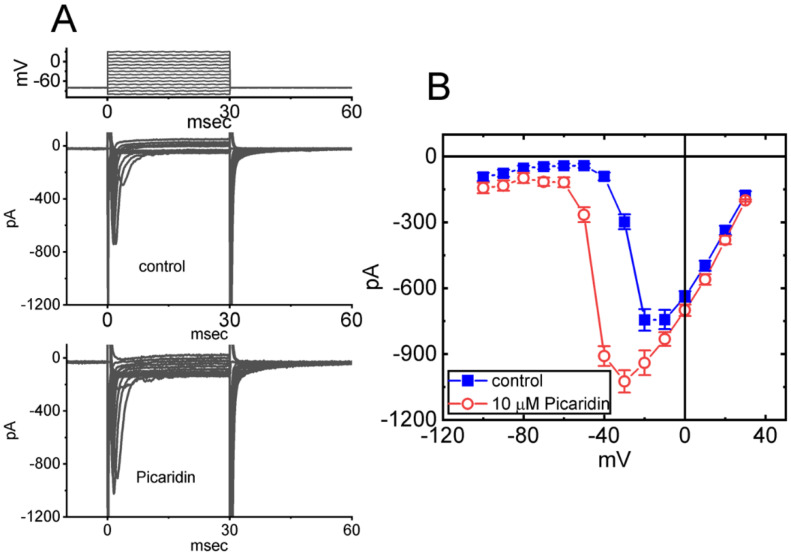
Mean current versus voltage (I–V) relationship of *I*_Na(T)_ caused by picaridin. Current amplitude (i.e., *I*_Na(T)_) was taken at the start of each abrupt depolarization. (**A**) Exemplar current traces achieved in the control period (upper) and during cell exposure to 10 μM picaridin (lower). The voltage-clamp protocol imposed is shown in the uppermost part. (**B**) Steady-state I–V relationship of *I*_Na(T)_ without (blue filled squares) and with (red open circles) cell exposure to 10 μM picaridin (mean ± SEM; n = 8 for each point). Note that cell exposure to picaridin depressed the amplitude of *I_Na_*_(T)_; moreover, the overall steady-state I–V relationship of the current was shifted to more negative potentials in its presence.

We next examined the perturbations of this compound on *I*_Na(T)_ measured at various membrane potentials. As depicted in Figure 2, a steady-state I–V relationship of *I*_Na(T)_ with or without the picaridin presence was constructed in these cells. The application of 10 μM picaridin resulted in a striking increase in the amplitude of *I*_Na(T)_ elicited by depolarizing steps. For example, as the examined cells were rapidly depolarized from −80 to −20 mV, the addition of picaridin (10 μM) noticeably raised the *I*_Na(T)_ magnitude from 744 ± 49 to 940 ± 56 pA (n = 7, *p* < 0.05). Moreover, the steady-state I–V relationship of *I*_Na(T)_ was found to be shifted to more negative potentials during GH_3_-cell exposure to 10 μM picaridin.

### 2.3. Characterization of Picaridin-Induced Modifications on the Steady-State Inactivation Curve of I_Na(T)_

**Figure 3:**
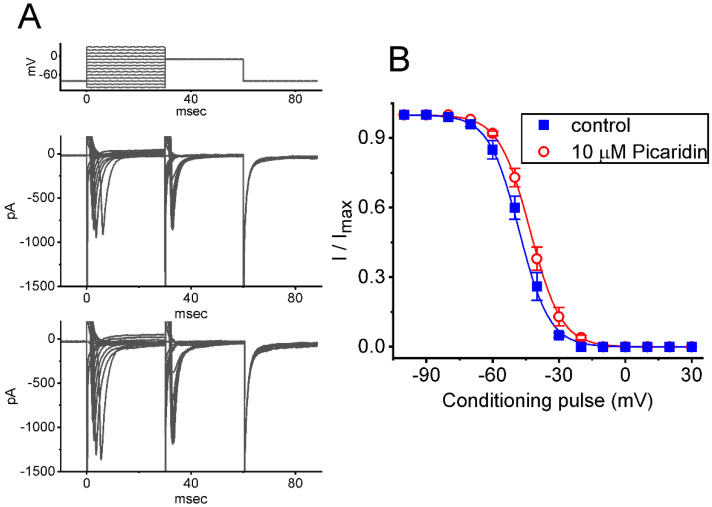
Modification by picaridin on steady-state inactivation curve of *I*_Na(T)_ identified from GH_3_ cells. This set of experiments was undertaken in situations where conditioning voltage pulses with a duration of 30 ms to various potentials, ranging between −100 and +30 mV, were applied from a holding potential of −80 mV. Following each conditioning pulse, a test pulse to −10 mV for a duration of 30 ms was, subsequently, applied to evoke *I*_Na(T)_. An example of current traces obtained by a two-pulse protocol in the control period (upper) and during the exposure to 10 μM picaridin (lower) is illustrated in (**A**). The uppermost part is the voltage protocol given. (**B**) Quasi-steady-state inactivation curves of *I_Na_*_(T)_ obtained with the absence (blue filled squares) and presence (red open circles) of 10 μM picaridine (mean ± SEM; n = 7 for each point). The normalized amplitude of *I*_Na(T)_ (i.e., I/I_max_) was constructed against the different levels of conditioning potential, and the sigmoidal curves (blue and red lines) were fitted to the Boltzmann equation (see Materials and Methods).

To further characterize the stimulatory effect of picaridin on *I*_Na(T)_ in response to an abrupt depolarization pulse, we continued to examine the steady-state inactivation curve of the current by using a two-step voltage protocol. Figure 3 shows the steady-state inactivation curve of *I*_Na(T)_ acquired with or without exposure to picaridin (10 μM). In this series of experiments, a 30 ms conditioning pulse was given to various membrane potentials to precede the test pulse (30 ms in duration) to −10 mV from a holding potential of −80 mV. The interval between two sets of voltage pulses applied was about 1 min to allow complete recovery of *I*_Na(T)_. The relationship between the conditioning potentials and the normalized amplitudes of *I*_Na(T)_ with or without the picaridin addition were plotted and, then, appropriately fitted to a Boltzmann function, detailed under Materials and Methods. In control, *V*_1/2_ = −48 ± 2 mV, *q* = 3.5 ± 0.6 *e* (n = 7), whereas in the presence of 10 μM picaridin, *V*_1/2_ −43 ± 2 mV, *q* = 3.6 ± 0.6 *e* (n = 7). It meant, therefore, that the presence of this compound not only elevated the maximal magnitude of *I*_Na(T)_, but it also shifted the steady-state inactivation curve of *I*_Na(T)_ to more depolarized potential (i.e., in the rightward direction) by approximately 5 mV. However, we found no appreciable adjustment in the *q* (apparent gating charge) value of the inactivation curve in its presence. These results reflected that the presence of picaridin could increase the *I*_Na(T)_ magnitude in a voltage-dependent fashion in GH_3_ cells.

### 2.4. Picaridin-Induced Retardation in Cumulative Inhibition of I_Na(T)_ Inactivation during Rapid Depolarizing Stimuli

**Figure 4:**
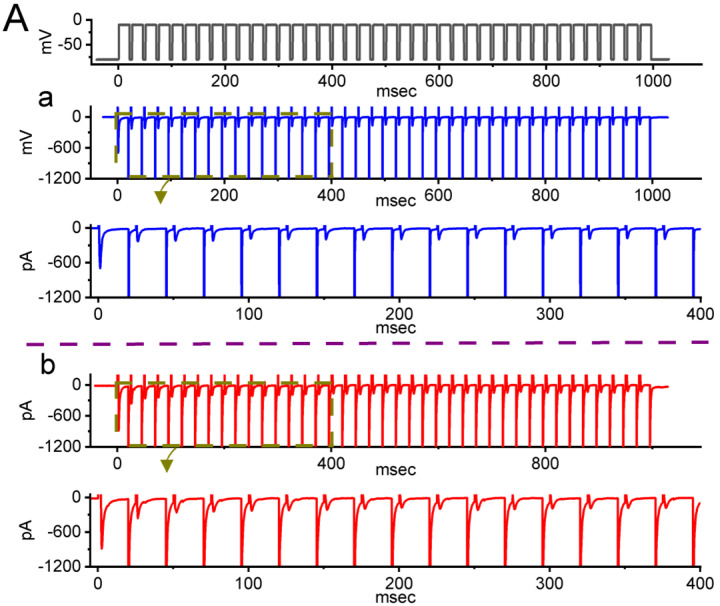

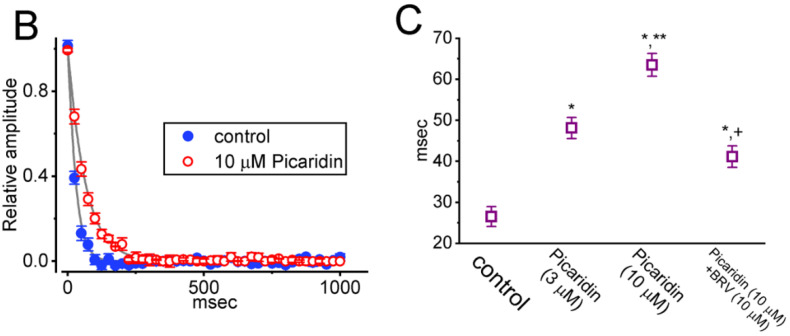
Effect of picaridin on *I*_Na(T)_ activated by a train of depolarizing pulses in GH_3_ cells. The voltage protocol employed consisted of 40 20-ms pulses (stepped to −10 mV) separated by 5 ms intervals at −80 mV for a total duration of 1 s. (**A**) Representative current traces taken during either the control period (**a**, absence of picaridin) or cell exposure to 10 μM picaridin (**b**). The voltage-clamp protocol delivered is illustrated atop the current traces. To provide single *I*_Na_ trace, the lower side in panels (**a**,**b**) denotes the expanded record from the brown broken box (with curve arrows) of the upper side in each panel. (**B**) The relationship of *I*_Na(T)_ versus the pulse train duration in the absence (blue filled circles) and presence (red open circles) of 10 μM picaridin (mean ± SEM; n = 7 for each point). The gray continuous lines on which the data points are overlaid was accurately fitted by a single exponential. Note that with cell exposure to picaridin, the time course of *I*_Na(T)_ inactivation during a train of depolarizing pulses slowed, together with an increase in time constant of the current inactivation. (**C**) Summary graph demonstrating the effect of picaridin (3 or 10 mM) and picaridin plus ranolazine (Ran) on the time constant of current decay in response to a train of depolarizing command voltage from −80 to −10 mV (mean ± SEM; n = 7 for each point). Current amplitude was measured at the beginning of each depolarizing pulse during depolarizing stimuli. * Signficantly different from control (*p* < 0.05), ** Significantly different from picaridin (3 μM) alone group (*p* < 0.05), and ^+^ Significantly different from picaridin (10 μM) alone group (*p* < 0.05).

It has been previously demonstrated that, prior to being activated during repetitieve short pulses, the inactivation of *I*_Na(T)_ can accumulate [27,38,39,40,41]. Therefore, in subsequent experiments, we proceeded to explore if the presence of picaridin could adjust the inactivation process of *I*_Na(T)_ during a train of membrane depolarizations. The tested cell was held at −80 mV, and the stimulus protocol, which was designed to consist of repetitive depolarization to −10 mV (20 ms in each pulse with a rate of 40 Hz for 1 s), was then imposed on it. In accordance with previous observations [38,40,41,42], as depicted in Figure 4A–C, during the control period (i.e., picaridin was not present), the process of *I*_Na(T)_ inactivation detected in GH_3_ cells was robustly evoked by a 1 s repetitive depolarization from −80 to −10 mV, and an evolving inactivation time constant of 26.5 ± 2.4 ms (n = 7) was yielded. That is, there was a sudden current decay with a single-exponential process. It is also noteworthy that during cell exposure of picaridin at a concentration of 3 or 10 μM, the time constant of *I*_Na(T)_ decay evoked by the same train of depolarizing voltage pulses was, respectively, raised to 48.1 ± 2.6 ms (n = 7, *p* < 0.05) or 63.5 ± 2.8 ms (n = 7, *p* < 0.05), in addition to a considerable increase in *I*_Na(T)_ amplitude. Moreover, upon continued presence of 10 μM picaridin, a further application of brivaracetam (BRV, 10 μM) noticeably attenuated the picaridin-prolonged time constant of *I*_Na(T)_ inactivation during repetitive depolarizations to 41.1 ± 2.6 ms (n = 7, *p* < 0.05). BRV was recently reported to suppress *I*_Na(T)_ amplitude [43]. Overall, these results meant that, apart from the increase in *I*_Na(T)_ amplitude, with cell exposure to picaridin the decrease in the decaying of *I*_Na(T)_ in response to a 1-s train of depolarizing pulses (i.e., accumulative inactivation of *I*_Na(T)_) could become retarded in these cells.

### 2.5. Effect of Picaridin on the Recovery Time Course of I_Na(T)_ Inactivation after the Conditioning Train of Depolarizing Stimuli

**Figure 5:**
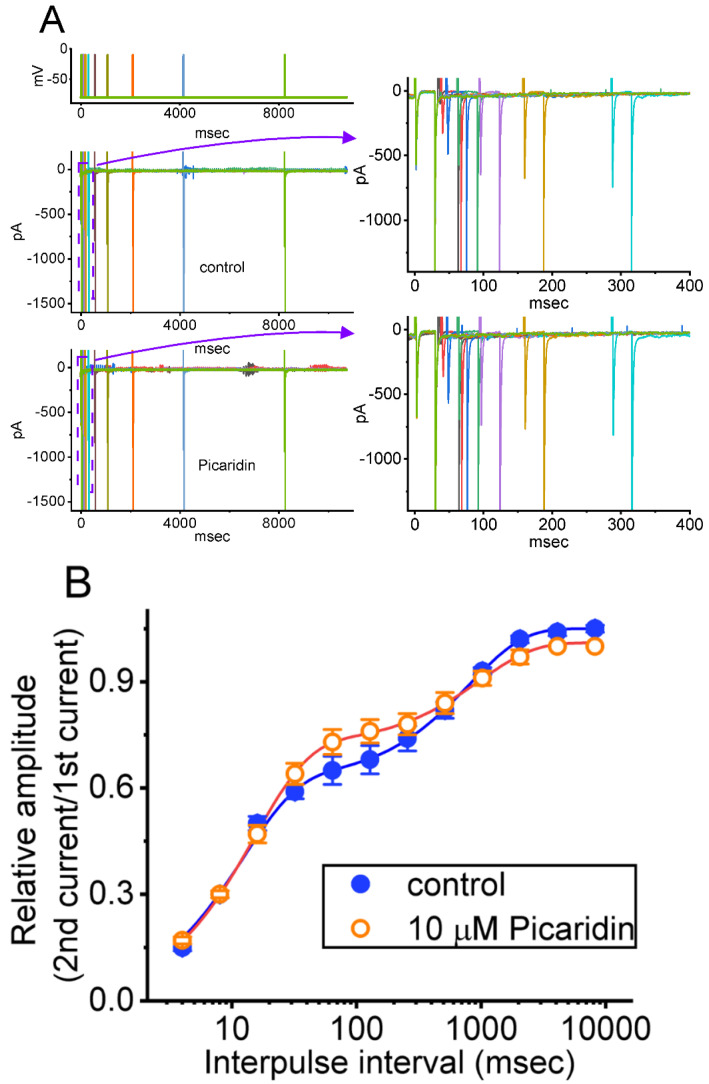
Modification by picaridin on the recovery of *I_Na_*_(T)_ inactivation evoked by varying inter-pulse intervals following the conditioning train of depolarizing pulses. In these measurements, we kept GH_3ˇ_cells bathed in Ca^2+^-free Tyrode’s solution, while the recording pipette was filled with a solution enriched with Cs^+^. The tested cells were depolarized from −80 to −10 mV for a duration of 30 ms, and subsequently variable inter-pulse durations with a geometric progression (indicated in the uppermost part) were imposed on them. (**A**) In our voltage-clamp protocol, the different colors indicated in current traces (lower) correspond with those in potential traces (upper). Exemplar current traces acquired in the absence (upper) and presence (lower) of 10 μM picaridin. Current traces in the right side show the expanded records from the purple dashed boxes with purple curve arrows in the left side for better illustrations, while the uppermost part is the voltage-clamp protocol applied. Brief inward deflection with a progressive rise with increasing inter-pulse interval showed the occurrence of *I*_Na(T)_ activated by short depolarizing pulse. (**B**) The relationships of the relative amplitude of *I*_Na(T)_ versus the inter-pulse interval acquired from the absence (blue filled circles) and presence (red open circles) of 10 μM picaridin (mean ± SEM; n = 8 for each point). The smooth curve obtained with or without the presence of picaridin was least-squares fitted with a two-exponential function, as described in Materials and Methods. Of note, the x-axis is given in a logarithmic scale.

The earlier investigations demonstrated a distinguishing type of recovery process from *I*_Na(T)_ inactivation, as elicited by a train of conditioning depolarizing stimuli ([38,41,44]. In light of these previous studies, we next sought to investigate if such a recovery process as that emerging in *I*_Na(T)_ could be modified by adding picaridin. In this separate set of experiments, the preceding condition train given was composed of 40 20-ms pulses separated by 5 ms intervals at −80 mV for a duration of 1 s. Following such conditioning train, a two-step voltage protocol, where the recovery process was measured, was further imposed on the tested cells to evoke *I*_Na(T)_. That is, the *I*_Na(T)_ was initially induced by a 30 ms step from −80 to −10 mV, voltage was thereafter returned to −80 mV for a variable length of time in a geometric progression with a common ratio of 2 (Figure 5A). In keeping with previous investigations, the resulting recovery time course, which slowed inactivation of *I*_Na(T)_ in response to the conditioning depolarizing stimuli, was noticed to emerge largely in a biphasic manner (i.e., fast and slowly recovering pools), while the recovery time course of current inactivation with no preceding conditioning train pulse was fitted in a satisfactory way by a single exponential. However, when the conditioning train pulse stimulation was applied (Figure 5B), during the control period, the experimental data were optimally fit with a sum of two exponential functions, which was characterized by fast (τ_fast_) and slow time constants (τ_slow_). Additionally, with cell exposure to picaridin, the evolving values of both τ_fast_ and τ_slow_ were noted to rise, while the fraction in the fast component increased and that in the slow component decreased. Table 1 summarizes the values of numerical parameters used for estimation of nonlinear recovery time course in the absence and presence of picaridin (3 or 10 μM). The results allowed us to propose that, in GH_3_ cells, the *I*_Na_ activated by a conditioning train of pulse could cause a large fraction of Na_V_ channels to shift toward the slowly recovering pool, as demonstrated in previous studies [38,44]. Moreover, of interest, during exposure to picaridin, the fraction of the slowly recovering pool of Na_V_ channels during the conditioning train pulse became smaller, while that of the fast recovery pool of the channels tended to be greater; however, both τ_fast_ and τ_slow_ values of recovery time course rose in its presence.

### 2.6. Effect of Picaridin on the Strength of Voltage-Dependent Hysteresis (Hys_(V)_) of Persistent I_Na_ (I_Na(P)_) Elicited by an Upright Isosceles-Triangular V_ramp_

**Figure 6:**
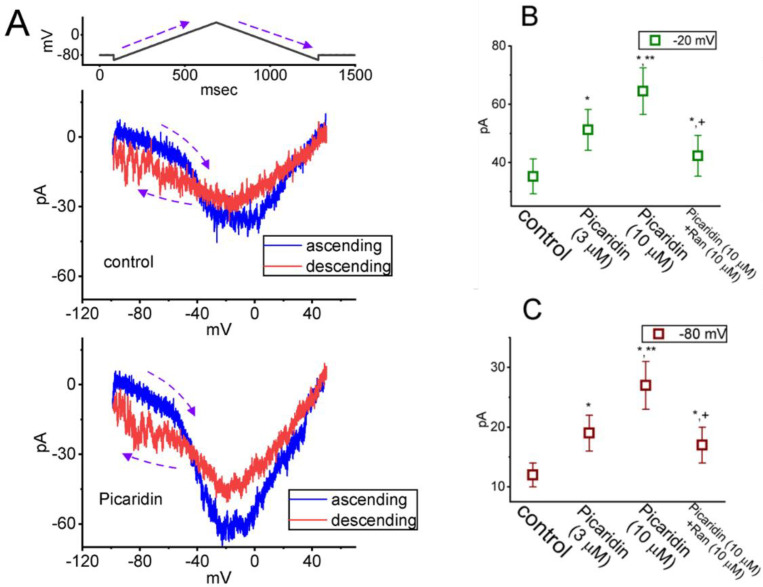
Modification by picaridin on voltage-dependent hysteresis (Hys_(V)_) of persistent *I*_Na_ (I_N(P)_) elicited by an upright isosceles-triangular ramp voltage (V_ramp_). The experiments were undertaken when the tested cell was voltage-clamped at −80 mV, and the V_ramp_ from −100 to +50 mV for a duration of 1.2 s (i.e., ramp speed of ±0.25 mV/ms) was applied to it. (**A**) Exemplar current traces acquired during the control period (upper part) and in the presence of 10 μM picaridin (lower part). The trajectory shown in blue or red color indicates current trace evoked at the ascending (up-sloping) or descending (down-sloping) limb of the upright triangular V_ramp_, respectively. The voltage protocol is illustrated atop current traces. The dashed curve arrows in each panel represent the direction of potential or current trajectory by which time goes. Summary graph showing the effects of picaridin (3 or 10 μM) and picaridin plus Ran on *I*_Na(P)_ amplitude activated by the up-sloping (ascending), (**B**) or down-sloping (descending), (**C**) limb of 1.2-s triangular V_ramp_ is, respectively, illustrated. The *I*_Na(P)_ amplitude at the up-sloping or down-sloping end of V_ramp_ was measured at −20 or −80 mV, respectively. Each point indicates the mean ± SEM (n = 8). * Significantly different from control (*p* < 0.05), ** Significantly different from picaridin (3 μM) alone group (*p* < 0.05), and ^+^ Significantly different from picaridin (10 μM) alone group (*p* < 0.05).

The Hys_(V)_ behavior of *I*_Na(P)_ has been recently demonstrated with a figure-of-eight (i.e., ∞-shaped) configuration as current trace was induced by an upright isosceles-triangular V_ramp_ [33,37,45,46]. Any modifications on *I*_Na(P)_ were expected to have a high impact on membrane excitability [47,48,49,50,51]. In this regard, we continued to study if the picaridin existence could modify the Hys_(V)_ strength in response to the upright isosceles-triangular V_ramp_. The measurements were undertaken in cells bathed in Ca^2+^-free Tyrode’s solution, and we filled up the electrodes with a solution containing Cs^+^. The tested cells were held at −80 mV and an upright isosceles-triangular V_ramp_, ranging between −100 and +50 mV for a duration of 1.6 s (i.e., ramp speed of ±0.25 mV/ms), was imposed on them. As demonstrated in Figure 6A,B in the control period, current traces in response to such triangular V_ramp_ evolved to exhibit two types of hysteretic loops (i.e., low- and high-threshold loops). The peak amplitude at low- and high-threshold loop appeared at around −80 and −20 mV, respectively, while current trajectory at low- and high-threshold loop occurred in a clockwise and anti-clockwise direction, respectively. As shown in Figure 6B, upon cell exposure to picaridin, the strength of *I*_Na(P)_’s Hys_(V)_ became overly increased. For example, upon cell exposure to this compound at a concentration of 10 μM, the *I*_Na(P)_ amplitudes at either high-threshold loop (i.e., at the level of −20 mV) or low-threshold loop (i.e., at the level of −80 mV), respectively, increased from 35 ± 6 to 65 ± 8 pA (n = 7, *p* < 0.05) or from 12 ± 2 to 27 ± 4 pA (n = 7, *p* < 0.05). Moreover, with continued exposure to 10 μM picaridin, subsequent addition of ranolazine (Ran, 10 μM) markedly attenuated *I*_Na(P)_ magnitude measured at −20 or −80 mV to 42 ± 7 (n = 7, *p* < 0.05) or 17 ± 3 pA (n = 7, *p* < 0.05), respectively. As such, it was reasonable to assume that the strength of *I*_Na(P)_’s Hys_(V)_ in response to long-lasting triangular V_ramp_ was susceptible to being enhanced in the presence of picaridin.

### 2.7. Picaridin-Mediated Increase of Resurgent I_Na_ (I_Na(R)_) Measured from GH_3_ Cells

**Figure 7:**
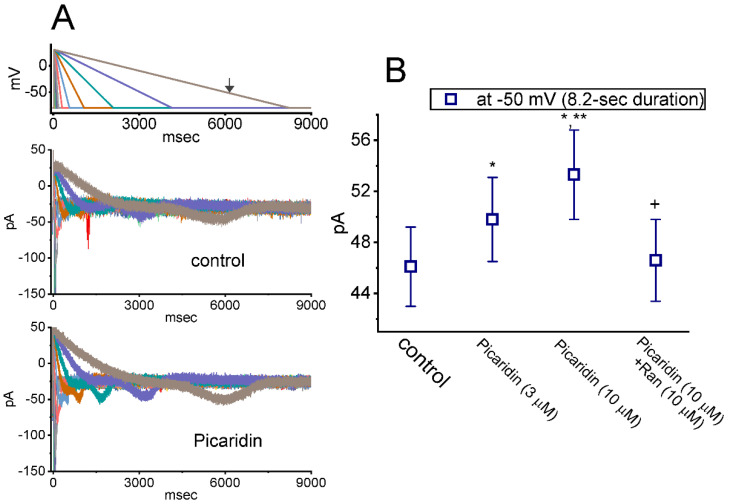
Augmentation by picaridin on resurgent I_Na_ (I_Na(R)_) evoked by the descending V_ramp_ with varying durations. The examined cell was held at the level of −80 mV, and a 30 ms depolarizing pulse to +30 mV was imposed. Following brief step depolarization, the down-sloping V_ramp_ from +30 to −80 mV, with varying duration in a geometric progression, was delivered to the cells. (**A**) In our voltage-clamp protocol, the different colors indicated in current traces (lower) correspond with those in potential traces (upper). Representative current traces obtained without (upper) or with (lower) the exposure to 10 μM picaridin. The uppermost part is the voltage-clamp protocol given, and the arrow indicates the level of −50 mV at which I_Na(R)_ amplitude evoked by V_ramp_ with a duration of 8.2 s was measured. (**B**) Summary graph demonstrating effects of picaridin (3 or 10 μM) and picaridin plus Ran on I_Na(R)_ amplitude (mean ± SEM; n = 7 for each point). Current amplitude was measured at the level of −50 mV during the 8.2-s descending V_ramp_. * This result is significantly different from control (*p* < 0.05), ** Significantly different from picaridin (3 μM) alone group (*p* < 0.05), and ^+^ Significantly different from picaridin (10 μM) alone group (*p* < 0.05).

*I*_Na(R)_ has been increasingly demonstrated to be responsible for burst generation and membrane excitability present in different excitable cells [32,41,51,52,53,54,55,56,57,58]. In this regard, we, thus, next wanted to study if the existence of picaridin produced any effects on *I*_Na(R)_ identified from these cells. The experiments used to measure *I*_Na(R)_ were, then, undertaken in situations where the examined cell was maintained at −80 mV and a brief depolarizing step to +30 mV for a duration of 30 ms was given to activate *I*_Na(T)_. Following the abrupt depolarizing step, a series of down-sloping V_ramp_’s from +30 to −80 mV, with varying durations in a geometric progression (with a common ratio of 2), was subsequently given to evoke *I*_Na(R)_ (Figure 7) [54]. The *I*_Na(R)_ amplitude evoked by such down-sloping V_ramp_ became progressively increased as cells were exposed to picaridin. For example, as GH_3_ cells were exposed to picaridin at a concentration of 3 or 10 μM, the *I*_Na(R)_ amplitude at the level of −50 mV evoked by an 8.2-s down-sloping V_ramp_ was respectively raised to 49.8 ± 3.3 pA (n = 7, *p* < 0.05) or 53.3 ± 3.5 pA (n = 7, *p* < 0.05) from a control value of 46.1 ± 3.1 pA (n = 7). Moreover, under continued exposure to picaridin, subsequent addition of Ran (10 μM) noticeably attenuated *I*_Na(R)_ amplitude at the same level of membrane potential to 46.6 ± 3.2 pA (n = 7).

### 2.8. Molecular Docking on Interaction between Na_V_1.7 Channel and Picaridin

**Figure 8:**
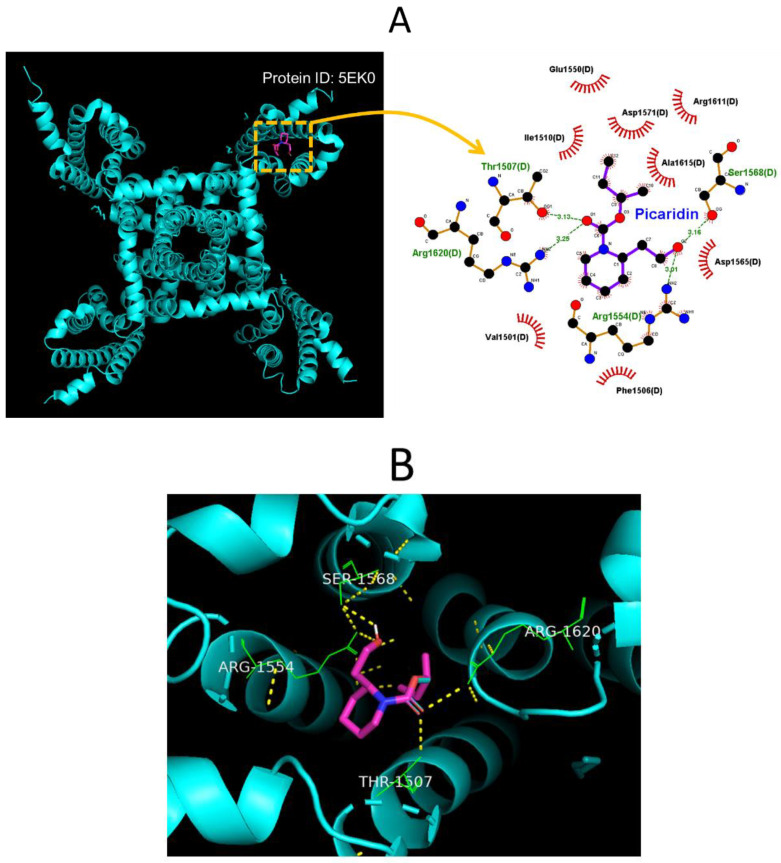
Results on molecular docking between the hNa_V_1.7 channel and the picaridin molecule. The protein structure of hNa_V_1.7 was obtained from RCSB (Research Collaboratory for Structural Bioinformatics) PDB (PDB ID: 5EK0), while the picaridin molecule was from PubChem (Compound CID: 125098 [3D conformer]). The structure about the hNa_V_1.7 channel was docked by the picaridin molecule with PyRx software (http://pyrx.sourceforge.io/) (accessed on 18 August 2022). (**A**) Diagram of the interaction between the hNa_V_1.7 channel and the picaridin molecule created from the LigPlot^+^ program (http://www.ebi.ac.uk/thornton-srv/software/LIGPLOT/) (accessed on 18 August 2022). Of notice, the red arcs on which small bars radiated toward the ligand (i.e., picaridin) represent hydrophobic interactions, while the green dotted lines residing in amino-acid residue (i.e., Thr1507(D), Arg1554(D), Ser1568(D), and Arg1820(D)) represent formation of hydrogen bonds. (**B**) Expanded graph for two-dimensional interaction sketch illustrating the predicted interactions of picaridin with residues (green color) in which hydrogen bonds are formed (indicated in yellow dotted line). The center in the diagram indicates the picaridin molecule.

In this study, we also continued to explore how the protein of the hNa_V_1.7 channel was optimally docked with the picaridin molecule, by using PyRx software. The protein structure of hNa_V_1.7 was taken from RCSB PDB (PDB ID: 5EK0) [59]. The predicted docking sites of the picaridin molecule, with which the amino-acid residues can interact, are presented in Figure 8A,B. The picaridin molecule was noted to form hydrophobic contacts with certain amino-acid residues, such as Val1501(D), Phe1506(D), Ile 1510(D), Glu1550(D), Asp1565(D), Arg1611(D), and Ala1615(D). The atoms in the picaridin molecule also have several hydrogen bonds with residue Thr1507(D) at a distance of 3.13 Å, Arg 1554(D) at 3.01 Å, Ser1568(D) at 3.16 Å, and Arg1620(D) at 3.25 Å, respectively (Figure 8A). The results of molecular docking hinted at the possibility that, apart from the ability of this compound to be active in targeting the odorant binding protein 1 (OBP1) [20,22], the picaridin molecule could potentially dock to the transmembrane region (position: 1597–1613) of hNa1.7 channel (PDB: 5EK0) with a binding affinity of −5.2 kcal/mol.

## 3. Discussion

In the present work, we provided evidence to show that the presence of picaridin, known to exert a repellent effect on insects, was able to exert stimulatory action on *I*_Na(T)_ and *I*_Na(L)_ seen in pituitary GH_3_ cells. The *I*_Na(L)_ in response to brief step depolarization was stimulated to a greater extent than the *I*_Na(T)_. Consequently, effective EC_50_ values needed for picaridin-stimulated *I*_Na(T)_ and *I*_Na(L)_ in these cells were yielded as 32.7 and 2.8 μM, respectively. The overall *I–V* relationship of *I*_Na(T)_ was shifted to a hyperpolarized potential by approximately 20 mV during exposure to picaridin, whereas the quasi-steady-state inactivation curve of *I*_Na(T)_ was shifted to a more depolarized potential by about 5 mV, being devoid of changes in the gating charge of the curve in its presence. The decaying time course of *I*_Na(T)_ activated during pulse train stimulation became noticeably slowed in the presence of this compound. Moreover, further addition of brivaracetam (BRV) attenuated picaridin-induced increase of *I*_Na(T)_’s decaying time constant responding to rapid membrane depolarizations. The recovery time course of *I*_Na(T)_ during the preceding conditioning pulse train was also strikingly altered upon cell exposure to this agent. When cells were exposed to this agent, the Hys_(V)_ magnitude in response to the upright isosceles-triangular V_ramp_ became strikingly augmented. The *I*_Na(R)_ magnitude in response to the descending V_ramp_ following initial brief depolarization was greatly enhanced by adding picaridin. Together, the activity of Na_V_ channels inherent in excitable cells (e.g., GH_3_ cells) may confer susceptibility to perturbations by picaridin or other structurally similar compounds; although, unlike other piperidine derivatives [28,30,32], this agent was observed to induce an excitatory action on *I*_Na_. The differential stimulation by picaridin of *I*_Na(T)_ and *I*_Na(L)_ is important and it may, thus, be responsible for its modulation of the electrical behaviors of excitable cells [3,60,61], presuming that the findings appear in vivo.

In the present study, the time-dependent decline of *I*_Na(T)_ activated during a 40 Hz train of depolarizing voltage steps, the protocol of which consisted of 20 ms pulses delivered from −80 to −10 mV at a rate of 40 Hz for a total duration of 1 s, was robustly detected in an exponential fashion, strongly indicating that there is marked use dependence of *I*_Na(T)_ elicited by repetitive depolarizations, as stated previously [39,40,41,42]. Moreover, such exponential decrease in *I*_Na(T)_ activated during pulse train stimulation was noted to become overly blunted with cell exposure to picaridin. Therefore, it is reasonable to assume that the existence of picaridin results in a gain-of-function change perturbed by the slowed time course of *I*_Na(T)_ inactivation, and that picaridin-induced increase of measured *I*_Na(T)_ is concurrently associated with conceivable use-dependent attenuation of *I*_Na(T)_, elicited as either high-frequency firing of action potentials or rapid repetitive stimuli occurring [38,42,62,63,64,65,66]. 

In this work, we also revealed that GH_3_-cell exposure to picaridin could cause the modifications on the recovery of *I*_Na(T)_ inactivation evoked by a train of conditioning depolarizing stimuli. Of note, under our experimental observations, the inactivation recovery process of measured *I*_Na(T)_, following the conditioning membrane depolarizations, was found to consist of two different fractions of current recovery, i.e., fast and slow recovering pool of *I*_Na(T)_. In other words, in agreement with earlier observations [38,41,44,67], the *I*_Na(T)_ activated by a conditioning train of pulses can lead to a certain fraction of Na_V_ channels to shift toward the slowly recovering pool during the current recovery process. As a result, there appears to be two distinct fractions of recovering pool (i.e., fast and slow) following the conditioning rapid membrane depolarization [38,68]. Furthermore, as summarized in Table 1, upon continued exposure to picaridin, the fraction of the fast recovery pool of Na_V_ channels observed under these experimental conditions tended to become greater, while that in the slow recovery pool of the channel was smaller. 

Work in our laboratory explicitly demonstrated the non-equilibrium Hys_(V)_ behavior of *I*_Na(P)_ activated by the upright isosceles-triangular V_ramp_, strongly indicating the striking voltage dependence of double V_ramp_-evoked *I*_Na(P)_ [49]. The experimental results also revealed two distinct types of measured Hys_(V)_ loops, which is reminiscent of the dynamics of the Lorenz-like system (i.e., figure-of-eight configuration) [69]. In other words, the Hys_(V)_ motion of the current as a function of time was noted to move in both counterclockwise and clockwise directions. One is a high-threshold counterclockwise loop with a peak of −20 mV, while the other is a low-threshold clockwise loop with a peak falling at around −80 mV. Furthermore, the exposure to picaridin was noted to accentuate Hys_(V)_’s strength of *I*_Na(P)_ responding to triangular V_ramp_. In continued exposure to picaridin, further addition of ranolazine markedly resulted in an attenuation of picaridin-mediated increase of Hys_(V)_ strength seen in GH_3_ cells. Ranolazine has been previously demonstrated to be an inhibitor of *I*_Na(L)_ [28,29,33,34]. Findings from the current observations, therefore, unveiled that the double V_ramp_-induced *I*_Na(P)_ resulted in striking Hys_(V)_ changes in voltage dependence and that such strength can be evidently enhanced by adding picaridin. 

In our study, the *I*_Na(R)_ in response to varying durations of repolarizing voltage was robustly detectable [32,41]. Of additional note, when cells were continually exposed to picaridin, the magnitude of *I*_N__a__(R)_ became overly increased. Moreover, the subsequent addition of ranolazine, still during continued exposure to picaridin, was able to attenuate the *I*_Na(R)_ magnitude, especially at the level of −50 mV. In accordance with previous reports showing that the *I*_Na(R)_ magnitude may alter the *I*_Na(T)_ inactivation during high-frequency stimulation [32,51,52,53,55,56,57,58,63,68], the current investigations allowed us to indicate that the existence of picaridin may perturb the electrical behaviors (e.g., aberrant and rapid firing of action potentials) residing in varying excitable cells.

## 4. Materials and Methods

### 4.1. Chemicals, Drugs, REAGENTS and Solutions Used in This Work

Zingerone Picaridin (icaridin, KBR 3023, Bayrepel^TM^, saltidin^®^_,_ 1-piperidinecarboxylic acid 2-(2-hydroxyethyl)-1-methylpropylester, hydroxyethyl isobutyl piperidine carboxylate [INCI], butan-2-yl 2-(2-hydroxyethyl)piperidine-1-carboxylate [IUPAC], C_12_H_23_NO_3_) were acquired from MedChemExpress (MCE^®^; Everything Biotech, New Taipei City, Taiwan). Ranolazine (Ran), tefluthrin, tetraethylammonium chloride (TEA), and tetrodotoxin (TTX) were obtained from Sigma-Aldrich (Genechain, Kaohsiung, Taiwan), while brivaracetam (BRV) was kindly provided by UCB (Union Chimique Belge) Pharm (PRA Health Sciences, Taipei, Taiwan). To protect picaridin from being degraded by light, stock solution containing this compound was wrapped with aluminum foil, and it was then kept under −20 °C for long-term storage. Unless specified otherwise, fetal calf serum, horse serum, culture media (e.g., Ham’s F-12 medium), L-glutamine, and trypsin/EDTA were mostly acquired from HyClone^TM^ (Genechain), whereas other chemicals or reagents were acquired from Sigma-Aldrich or Merck (Genchain) and were of laboratory grade. 

The standard external solution (i.e., normal Tyrode’s solution) had the composition (in mM): NaCl 136.5, CaCl_2_ 1.8, KCl 5.4, MgCl_2_ 0.53, glucose 5.5, HEPES 5.5, and the pH was adjusted to 7.4 by adding NaOH. To record ionic flowing through K^+^ currents, the electrode was filled up with the internal pipette solution containing (in mM): K-aspartate 130, KCl 20, KH_2_PO_4_ 1, MgCl_2_ 1, EGTA 0.1, Na_2_ATP_3_, Na_2_GTP 0.1, and HEPES 5, and the pH was adjusted to 7.2 by adding KOH. However, to measure *I*_Na_, K^+^ ions in the pipette solution were replaced with equimolar Cs^+^ ions, and the pH was adjusted to 7.2 with CsOH. The twice-distilled water used for the current experiments was deionized through Millipore-Q^®^ system (Merck, Tainan, Taiwan).

### 4.2. Cell Preparations

Clonal pituitary (GH_3_) somatolactotrophs, originally acquired from the Bioresources Collection and Research Center ([BCRC-60015, http://catalog.bcrc.firdi.org.tw/BcrcContent?bid=60015] (accessed on 18 August 2022), Hsinchu, Taiwan), were cultured in Ham’s F-12 medium, which was supplemented with 15% heat-inactivated horse serum (*v*/*v*), 2.5% fetal calf serum (*v*/*v*), and 2 mM L-glutamine. They were grown at 37 °C in monolayer cultures in 50-mL plastic culture flasks in a humidified environment of 5% CO_2_/95% air. We performed electrical recordings 5 or 6 days after GH_3_ cells underwent subculture (60–70% confluence).

### 4.3. Electrophysiological Measurements

Shortly before the measurements, we carefully dispersed cells by using 1% trypsin-EDTA solution, and quickly put a small portion of the suspension containing clumps of cells into a recording cubicle fixed on the stage of an inverted microscope (DM-IL; Leica; Major Instruments, Tainan, Taiwan). We maintained cells at room temperature (20–25 °C) in HEPES-buffered normal Tyrode’s solution, the ionic compositions of which are stated above. The patch electrodes used to record were constructed from melting point capillaries made of Kimax^®^-34500-99 glass with 1.5–1.8 mm outer diameter (Sigma-Aldrich, Genechain, Kaohsiung, Taiwan) by using a PP-83 vertical puller (Narishige, Major Instruments). When filled with different internal solutions described above, the recording pipettes had tip resistances of 3–5 MΩ. The electrodes were held in place by micromanipulators. We recorded ionic currents in the whole-cell configuration by making some small modifications to the original patch-clamp technique with an RK-400 patch amplifier (Bio-Logic, Claix, France), as described elsewhere [31,32,70,71]. The GΩ-seals, which were made in an all-or-nothing fashion, led to a significant improvement in the signal-to-noise ratio. The liquid junction potential, which occurred due to different compositions between external and internal solutions, became nulled shortly before the GΩ-seal formation was made, and the whole-cell data were then corrected.

### 4.4. Data Recordings and Processing

The signal output (i.e., potential and current traces) was monitored and digitized online at 10 kHz or more in an ASUS ExpertBook laptop computer (Yuan-Dai, Tainan, Taiwan). For analog-to-digital (A/D) and digital-to-analog (D/A) conversion, a Digidata^®^ 1440 A device interfaced to the computer was controlled by pCLAMP^TM^ 10.6 program run under Microsoft Windows 7 (Redmond, WA, USA) [70,71]. We low-pass filtered current signals at 2 kHz by using a FL-4 four-pole Bessel filter (Dagan, Minneapolis, MN, USA). The voltage-clamp protocols, consisting of manifold rectangular or ramp waveforms, were specifically designed, and they were then delivered to the tested cells through D/A conversion.

### 4.5. Data Analyses for Whole-CELL Ionic Currents

To determine concentration-dependent stimulation of picaridin on the amplitude of *I*_Na(T)_ or *I*_Na(L)_, we placed GH_3_ cells into Ca^2+^-free Tyrode’s solution. During the measurements, we maintained each cell at −100 mV, and a short depolarizing pulse to −10 mV for 30 ms was delivered to evoke *I*_Na_. The *I*_Na(T)_ or *I*_Na(L)_ magnitudes were then, respectively, measured at the beginning- or end-pulse of 30-ms depolarizing step when cumulative application of different picaridin concentrations was given. The amplitude of *I*_Na(T)_ obtained in the presence of picaridin at a concentration of 100 μM was taken to be 100% and current magnitudes during exposure to varying concentrations of this compound were subsequently compared. The concentration-dependent stimulation by picaridin of *I*_Na(T)_ or *I*_Na(L)_ residing in GH_3_ cells was determined by fitting experimental data set to a modified Hill function [31,32]. That is:

$$percentage\;increase\;\left(\%\right)=\left(E_{max}\times {\left[picaridin\right]}^{n_{H}}\right)/\left(EC_{50}^{n_{H}}+{\left[picaridin\right]}^{n_{H}}\right)$$

where [*picaridin*] = the picaridin concentration given; *n_H_* = the Hill coefficient (i.e., coefficient for cooperativity); *EC*_50_ = the concentration required for a 50% stimulation of *I*_Na(T)_ or *I*_Na(L)_ amplitude activated by abrupt depolarizing step; and *E_max_* = maximal stimulation of *I*_Na(T)_ or *I*_Na(L)_ caused by the picaridin presence.

To evaluate picaridin-mediated adjustments on the quasi-steady-state inactivation of *I*_Na(T)_, the relationship of the normalized amplitude of *I*_Na(T)_ versus the conditioning potential obtained with or without the presence of 10 μM picaridin was established, and the data set was then fitted optimally to a Boltzmann function on the following form:

$$I=I_{max}/\left\{1+exp\left[\left(V-V_{\frac{1}{2}}\right)RT/qF\right]\right\}$$

where *I_max_* = the maximal *I*_Na(T)_ magnitude acquired in the absence or presence of picaridin (10 μM) at the conditioning membrane potential of −100 mV; *V* = the conditioning potential; *V*_1/2_ = the potential required for half-maximal activation (i.e., midpoint potential); *q* = the apparent gating charge needed; *F* = Faraday’s constant; *R* = the universal gas constant; *T* = the absolute temperature; and *RT/F* = 25.2 mV.

By use of a two-step voltage protocol with variable interpule intervals in a geometric progression, the recovery time course of *I*_Na_ from block activated in response to the 1-s conditioning pulse train was optimally constructed, and the results acquired with or without the addition of picaridin were thereafter drawn by plotting the *I*_Na(T)_ amplitude (normalized with respect to the steady-state amplitude activated at 0.1 Hz). A basic assumption of the analyses was that the recovery time course of the current measured under these experimental conditions could be described in a satisfactory manner by exponential function [38,67,72]. Due to the recovery time course in GH_3_ cells exhibiting a rapidly rising recovery phase, followed by a late slow phase, there should be at least two underlying exponential terms. Therefore, the data points showing recovery time course with or without exposure to picaridin were least-squares fitted to the exponential function with biexponential process. That is:

(1) y=A×(1−e−tτfast)+B×(1−e−tτslow)

where *y* is the relative *I*_Na(T)_ amplitude at time *t*, *A* or *B* is the relative amplitude of each exponential component, and *τ_fast_* or *τ_slow_* is the fast or slow time constant in the recovery of *I*_Na_ block, respectively. The experimental results were optimally analyzed and they are summarized in Table 1.

### 4.6. Curve-Fitting Approximations and Statistical Analyse

Curve-fitting (linear or nonlinear) to the experimental data set applied was fitted by use of manifold analytical tools, that included the Microsoft “Solver” embedded into Excel^®^ 2022 (Microsoft) and OriginPro^®^ 2022b program (OriginLab^®^; Microcal; Scientific Formosa, Kaohsiung, Taiwan) [40,41]. The electrophysiological data are presented as the mean ± standard error of the mean (SEM). The size of independent observations (n) is indicated in cell numbers collected during the experiments. The data distribution obtained was found to satisfy the tests for normality. For comparisons between the two different groups, we performed Student’s *t*-tests (for paired or unpaired samples). However, for more than two different groups, analysis of variance (for one- or two-way ANOVA) with or without repeated measure was followed by a post-hoc Fisher’s least-significance difference test, which was applied where appropriate to evaluate individual differences among multiple groups of data. A statistical significance (indicated with *, ** or + in the figures) was considered when *p* < 0.05.

## 5. Conclusions

The emerging data reveal that picaridin, known to be an insect and acarid repellent in the piperidine chemical family, leads to profound stimulation in *I*_Na_ in concentration-, time-, voltage-, frequency-, and Hys_(V)_-dependent manners. The precise ionic mechanism of picaridin actions on *I*_Na_ needs to be further clarified. The GH_3_ cell line used was demonstrated to express the α-subunits of Na_V_1.1, Na_V_1.2, Na_V_1.3, and Na_V_1.6, as well as the β1 and β3-subunits of Na_V_ channel [24,73]. Moreover, the results of molecular docking also prompt us to predict the effectiveness of picaridin in binding to certain amino-acid residues intrinsically in the hNa_V_1.7 channel, by forming either hydrophobic contacts or hydrogen bonds. The present data obtained from GH_3_ cells, thus, sheds light on the evidence demonstrating the effectiveness of picaridin in adjusting specific ionic currents (e.g., *I*_Na(T)_, *I*_Na(L)_, *I*_Na(P)_ and *I*_Na(R)_). These results may be linked to the additional, but important, actions of picaridin, or other structurally similar compounds, which are potentially useful in pharmacological or toxicological applications in different excitable cells occurring in vivo.

## Figures and Tables

**Table 1:** Summary of results showing the parameter values for effects of picaridin on the recovery of the *I*_Na_ block during the preceding train pulse. These parameters are elaborated in detail in Materials and Methods. All values are mean ± SEM. * Significantly different from controls (*p* < 0.05).

	N	τ_fast_ (ms)	τ_slow_ (ms)	A	B
Control	8	12.1 ± 0.5	801 ± 12	0.62 ± 0.04	0.43 ± 0.02
Picaridin (3 μM)	8	13.9 ± 0.6 *	893 ± 14 *	0.67 ± 0.04 *	0.36 ± 0.02 *
Picaridin (10 μM)	8	15.1 ± 0.7 *	976 ± 15 *	0.72 ± 0.04 *	0.29 ± 0.02 *

## Data Availability

The original data is available upon reasonable request to the corresponding author.

## Abbreviations

BRV	brivaracetam
EC_50_	concentration required for half-maximal stimulation
Hys_(V)_	voltage-dependent hysteresis
*I*_Na(L)_	late Na^+^ current
*I*_Na(T)_	transient (peak) Na^+^ current
Na_V_ channel	voltage-gated Na^+^ channel
Ran	ranolazine
SEM	standard error of mean
TEA	tetraethylammonium chloride
τ_inact(S)_	slow component in inactivation time constant
V_ramp_	ramp voltage
TTX	tetrodotoxin
V_ramp_	ramp voltage
